# The impact of market integration on arranged marriages in Matlab, Bangladesh

**DOI:** 10.1017/ehs.2022.54

**Published:** 2022-12-12

**Authors:** S.B. Schaffnit, A. E. Page, R. Lynch, L. Spake, R. Sear, R. Sosis, J. Shaver, N. Alam, M.C. Towner, M. K. Shenk

**Affiliations:** 1 Pennsylvania State University, State College, PA, USA; 2 London School of Hygiene and Tropical Medicine, London, UK; 3 University of Otago, Dunedin, New Zealand; 4 Binghamton University, Binghamton, NY, USA; 5 University of Connecticut, Storrs, CT, USA; 6 International Centre for Diarrheal Disease Research, Dhaka, Bangladesh; 7 Oklahoma State University, Stillwater, OK, USA

**Keywords:** arranged marriage, love marriage, arranged love marriage, market integration, Bangladesh

## Abstract

Success in marriage markets has lasting impacts on women's wellbeing. By arranging marriages, parents exert financial and social powers to influence spouse characteristics and ensure optimal marriages. While arranging marriages is a major focus of parental investment, marriage decisions are also a source of conflict between parents and daughters in which parents often have more power. The process of market integration may alter parental investment strategies, however, increasing children's bargaining power and reducing parents’ influence over children's marriage decisions. We use data from a market integrating region of Bangladesh to (a) describe temporal changes in marriage types, (b) identify which women enter arranged marriages and (c) determine how market integration affects patterns of arranged marriage. Most women's marriages were arranged, with love marriages more recent. We found few predictors of who entered arranged vs. love marriages, and family-level market integration did not predict marriage type at the individual level. However, based on descriptive findings, and findings relating women's and fathers’ education to groom characteristics, we argue that at the society-level market integration has opened a novel path in which daughters use their own status, gained via parental investments, to facilitate good marriages under conditions of reduced parental assistance or control.

## Graphical abstract

**Figure:**
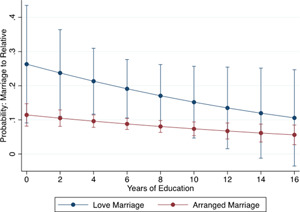

**Social media summary:** Market integration leads to a rise in love marriages in Bangladesh. Daughters rely less on parents’ involvement, use own status to facilitate marriages.

## Introduction

A successful marriage is one that is both optimally timed and joins suitable partners. Success on the marriage market has long-lasting implications for the fitness of women and their future children as played out through their health and socioeconomic status (Shenk, 2004, 2005, 2007; Shenk & Scelza, 2012; Boone, 1986; Kaplan, 1996; Kramer & Lancaster, 2010; Santhya, 2011; Schaffnit, Hassan et al., 2019; Shenk, Towner et al., 2016; Apostolou, 2010). Parents therefore invest heavily in facilitating advantageous unions for their children by using their resources or social connections to secure spouses who will provide desirable outcomes for their child, other children and the wider family. Parents also directly invest in their children to increase their desirability on the marriage market by, for example, saving for or negotiating financial transfers at the time of marriage (e.g. bride wealth or dowry) and/or investing in their children's education. Given the importance of marriage market outcomes for children and the unique position parents have to arrange advantageous matches, it is perhaps unsurprising that arranged marriages, i.e. marriages of children negotiated by parents and/or extended kin networks, are common across a majority of human societies (e.g. Shenk, 2017; Apostolou, 2007, 2010; Shenk, Towner et al., 2016; Walker et al., 2011; Murdock, 1949; Berghe, 1979).

### The behavioural ecology of arranged marriage

Although arranged marriage is common, parents' desire and ability to arrange children's marriages varies by socioecology (Apostolou, 2010, 2011; Shenk, Towner et al., 2016) and children's characteristics. For example, arranged marriages are most common in societies where wealth is heritable and/or transferred at marriage, and where family alliances play key roles in a family's economic or political importance (Shenk, 2017; Coontz, 2006; Lévi-Strauss, 1949). As such, while present in foraging and horticultural populations, arranged marriage practices are much more common in agropastoralist societies (Apostolou, 2010), where parents have a monopoly on heritable resources (e.g. land or animals) and are able to use their accumulated wealth and status through marriage negotiations or marriage payments to secure spouses for their children.

Patterns of arranged marriage are also gendered – daughters are more likely to experience arranged marriages than sons – owing to power imbalances between sexes, both in the parent–child and husband–wife dyads, and practical considerations. Women generally marry at younger ages compared with men, who often marry later, which is likely both a reflection and cause of their relative lack of power in arranging their own marriages. On the other hand, men are more likely to earn their own income, inherit from their parents (or inherit a larger share than their sisters), and otherwise control their own resources, and are thus more likely to arrange marriages for themselves through discussion with a potential bride's parents and/or family (Broude & Greene, 1983; Agey et al., 2021; Apostolou, 2007, 2010; Shenk, 2017). On a practical level within patrilocal societies, parents are generally more meticulous and involved in choosing a spouse for their daughters than sons because women leave home at marriage and the choice of spouse has a profound effect on the future life of the daughter and her children (e.g. Apostolou, 2010; Shenk, 2007); in contrast, a woman who marries a son will have a less consequential effect on the socioeconomic situation of the family she joins. For these reasons, as our paper focuses on a patrilocal context, we specifically focus on arranged marriages for women.

While parents’ participation in facilitating their daughters’ marriages is an important form of parental investment, it can also be a source of conflict. Just as daughters can benefit from advantageous marriages, parents and extended kin can benefit from financial transfers at the time of marriage, and strengthened social networks through endogamy and/or widened social networks through exogamy (Shenk, Towner et al., 2016; Chapais, 2008; Coontz, 2006; Lévi-Strauss, 1949). Certain features of women's marriages, including timing and spousal characteristics, also impact children's reproductive fitness and thus affect their parents’ fitness as well (Raj & Boehmer, 2013). Parents' and children's interests often align, but parents are expected to prefer marriages that advantage their whole family unit even when there is a potential cost to an individual child (Trivers, 1974; Schaffnit, Hassan et al., 2019). For example, parents may prefer their daughters to marry at younger ages or in times of familial economic need in order to optimise the timing and size of bridewealth or dowry payments (Corno & Voena, 2016; Schaffnit, Hassan et al., 2019). Parents may also prioritise marriages which build advantageous family alliances (Chagnon et al., 2017; Coontz, 2006; Lévi-Strauss, 1949), including, for example, prefering spouses from well-off or high-status backgrounds. In contrast, daughters may prefer to marry at older ages to benefit from prolonged investments in embodied capital and reduced risks associated with early reproduction (Apostolou, 2015). Evidence of disagreement between parents and their children over marriages abounds, especially regarding ideal partners and timing of marriage (Weissner, 2009; Agey et al., 2021). Yet not all parent–child disagreements are indicative of a true evolutionary conflict. For example, daughters often prioritise love and attraction to a husband more highly than their parents, potentially leading parents to select a partner who is less attractive to their daughter. While such a situation may result in disagreement between parents and daughter, if parents in fact select a spouse for their daughter based on more fitness-impacting traits, then parents may be acting in the fitness interests of their daughter, making the conflict superficial.

Whether arranged marriage systems are viewed as a source of conflict or as a form of parental investment – or most likely both – parents have historically had the upper hand over their children when it comes to marriage decisions (Voland, 1998; Boone, 1986). Parents are usually physically larger (when their children are young and/or female; Apostolou, 2007), and generally control more resources and hold greater social sway than their children. As such, when conflicts do arise, parents are often in a better bargaining position – especially in contexts where parents control many economic and social resources and/or children marry at young ages (Alexander, 1974; Shenk, 2017; Weissner, 2009). In fact, even in the absence of outward disagreement between parents and children over marriage, the presence of arranged marriage systems and effective parental control over mate choice are indicative of parents having ‘won’ such conflicts, i.e. parents having systematically greater bargaining power than their children.

### Impact of market integration on arranged marriage systems

Market integration may, however, fundamentally alter parent–child dynamics in marriage markets. Market integration is a process that entails a shift away from agriculture to occupations less directly related to subsistence, e.g. market work, or education-based employment, but it also encompasses wider economic and societal changes that occur alongside this process (Mattison et al., 2022; Lu, 2007). Evolutionary anthropologists have argued that market integration results in increased parental investment, particularly in embodied capital via education (Kaplan, 1996; Lawson & Mace, 2011), a phenomenon that results in a levelling of embodied capital differences between parents and children. Shifts from subsistence to market-based economies (i.e. market integration) thus increase children's bargaining power within the parent–child dyad. This is because as children become educated and more involved in generating wealth for themselves and their families, they gain influence within the family, thereby reducing relative parental influence and increasing their ability to make their own marriage choices even in the face of parental opposition. This has been shown in semi-rural Tanzania, where parents often attempt to control their children's, particularly daughters’, sexual and marital behaviours in order to secure advantageous bride wealth payments and social benefits (Wamoyi et al., 2011a). However, recent increases in children's education and economic contributions to their family unit have resulted in reductions in parental control and increased decision-making power among children (Wamoyi et al., 2011b). Among Ju/’hoansi foragers of South Africa, parents are highly motivated to control each step in the marriage process of their children, frequently desiring their children to marry at young ages to politically advantageous spouses (Weissner, 2009). Through physical coercion of children (e.g. beating) or threats of suicide, parents were historically successful in enforcing their desires despite resistance from their children (e.g. running away). However, through government schemes in the 1960s and 1970s the Ju/’hoansi were settled, and wage labour amongst young men and education for girls increased. These changes altered parent–child relationships and the dynamics of marriage. Young people now control their own marriages and these marriages tend to occur at older ages than they did in previous decades (Weissner, 2009). Furthermore, the fertility declines that often accompany market integration may also alter bargaining dynamics in marriage negotiations as extended kin networks disperse to different cities or regions and shrink in size and influence (Colleran, 2020).

In addition to changes in parent–child bargaining dynamics, market integration and accompanying fertility decline also alter parental investment strategies such that parents often invest more heavily in fewer children (Lawson & Borgerhoff Mulder, 2016; Shenk, Towner, et al., 2013; Kaplan et al., 2002; Lawson & Mace, 2011). Market integration can also prompt increasing inequalities within communities and parents must increase their investments in their children – through education, health, marriage etc. – to ensure their future success (Shenk, Kaplan, & Hooper, 2016; Boone, 1986). Under such circumstances, parents' motivation to control children's marriages may remain high even as their ability to enforce their choice grows more limited in the face of the increasing participation of children in formal schooling, children's rising economic autonomy and the decoupling of the economic cooperation between parents and children (e.g. as children move away from family farms and trades and into the labour market). Thus, increasing parental investments owing to market integration reinforce children's already strengthened bargaining power. Further, such increases in parental investments and investments in embodied capital typically result in delays to marriage which again strengthen children's bargaining power in marriage decisions owing to their more advanced age and the economic productivity which accompanies it (Apostolou, 2011). Spousal choice in such contexts often moves from norms of parental choice towards norms of joint decision-making or even child choice (Shenk, 2017; Coontz, 2006), and direct parental investments in children (e.g. in their education, occupation, or income) often become the primary way that parents work to attract a desirable spouse (Shenk, 2004; Coontz, 2006).

### Study setting

In this study, we consider patterns of arranged marriage for women in Matlab, Bangladesh, a rural area experiencing rapid market integration. Currently, many residents of Matlab still participate in agriculture but do not own the land that they farm and most households engage in wage labour to supplement agriculture (Alam et al., 2017; Shenk, Towner et al., 2016). Recent decades have seen an expansion of both wage labour within Matlab and labour migration out of Matlab, whereby emigrants send remittances back to their families (ICDDR,B, 2018). Based on these ongoing changes in economy of households in Matlab, in this paper we operationalise family-level market integration using proxies derived from data on women's fathers’ occupation (Shenk, Towner, et al., 2013).

Arranged marriage is an ancient custom in South Asia, including Bangladesh, and has been normative regardless of the religion of the family or the type of marriage payment, e.g. dowry or bridewealth. While arranged marriages still account for the vast majority of marriages in Matlab, recent decades have seen increases in love and love–arranged marriages. Love matches are those in which a person selects and marries a spouse of their own choosing, sometimes without the approval of their family, while in a love–arranged marriage a person selects their spouse but their parents/relatives arrange the marriage – either because they approve of the spouse or for the sake of the family's reputation and prestige. Owing to sample size limitations, marriages that are not fully arranged will be collectively referred to as love marriages from here on, although they include both love matches and love–arranged marriages. In the cases where we distinguish between them, we will specify ‘purely love’ marriages to exclude love–arranged marriages.

Parents in Matlab use their own status to negotiate advantageous marriages for their daughters yet simultaneously invest heavily in daughters to secure a good match. Parents commonly invest in their daughters through education – with balances made between being educated to a high enough level and not postponing marriage too long (Shenk, 2004; Shahidul, 2014) – and through savings for dowry, that is payments flowing from a woman's family to her groom's family at the time of marriage. In Bangladesh and other areas of South Asia, dowry is a major form of parental investment, critical to the success of daughters on the marriage market (Shenk, 2004; Shahidul, 2014). The custom of dowry is ancient in the region, and while in the past it was typically practised mainly by wealthy land-owning or merchant families, in recent decades dowry has become normative in Bangladesh as in much of the rest of South Asia (Srinivas, 1984; Lankes et al., 2022). Many have argued that this spread of dowry is linked to economic development/market integration (Shenk, 2004; Srinivas, 1984) through education and savings for dowry. In this region dowry payments are common across most marriages, but are less likely to occur in love marriages, particularly those lacking parental approval.

### Research questions and predictions

Using data from, 2010 collected in Matlab Bangladesh, we address three research questions, the latter two with associated predictions:

1. What are the temporal changes in arranged vs. love marriages for women?
2. Which characteristics of women are associated with arranged vs. love marriages?
  **[P1]** Women who had arranged marriages will have higher status mates than those who had love marriages. This is because we expect that parents will be able to use their relatively greater wealth and/or status to attract a higher status husband for their daughters than a daughter would on her own. We thus further expect that, independent of marriage type, parental ability to attract a desirable spouse for their daughter will be an important predictor of the husband's status. This prediction is consistent with the hypothesis that arranged marriage is a means of parental investment, and that parental resources are positively correlated with spouse quality for children (Voland, 1998).
  **[P2]** Love marriages will take place at later ages because early marriages, especially when closely followed by first births, can be costly for girls and their children (Kramer & Lancaster, 2010). While parents generally seek to avoid harm to their children, they may experience conflicting interests with an individual child; parents sometimes benefit from earlier marriages through lower dowry costs (in areas where younger women are seen as more biddable or chaste), or through positive effects of the marriage of a child on the family's economic status or social connections, and thus may be less risk averse than their daughters, who may prefer to marry later (Schaffnit & Lawson, 2021). Such a pattern, however, could arise owing to delays in marriage related to either (a) the opportunities to meet prospective spouses afforded by extended education or (b) the fact that more educated daughters have greater bargaining power when the marriage finally occurs. Moreover, this relationship may be moderated by whether the marriage is to a relative or not. If love marriages are more frequently to cousins (e.g. Ghimire et al., 2006; Shenk, Towner et al., 2016), especially for women with low levels of education, then these marriages may take place at younger ages both because delays will not be made for education and because women are acquainted with their cousins from a young age.
3. How do aspects of market integration relate to patterns of arranged and love marriages?
  **[P3]** Women from families that are less market integrated will be more likely to have had an arranged marriage than women from more market integrated families. Specifically, we expect that love marriages will be more common in market-integrated families because daughters are likely to be better educated, marry at later ages, and have more opportunities to meet prospective husbands (e.g. Shenk, Starkweather et al., 2013; Shenk, Towner et al., 2016).
  **[P4]** Women with higher education who enter love marriages will be less likely to marry a relative, while women with lower education who enter a love marriage will be more likely to marry a relative. Within Matlab and other parts of South Asia, love marriages are also likely to be to relatives because socialising between unrelated, unmarried men and women is restricted and women are more likely to interact and thus fall in love with male relatives than with unrelated men (Ghimire et al., 2006; Shenk, Towner et al., 2016). Further, both women and their parents may prefer marrying relatives when entering a love marriage because there are fewer unknowns about their future in-laws (Maqsood, 2021). Whether love marriages are more likely to be with a relative or not could therefore depend on both exposure to non-relatives (such as through secondary or post-secondary education) and the motivation to expand one's family network by marriage to a non-relative, which has become a prominent motivation in contemporary Matlab (Shenk, Towner et al., 2016). Compared with women with fewer years of education, women with more education may have greater bargaining power to pick a spouse, and more opportunities to meet unrelated men than those with lower education, and may therefore favour marrying out of their family in order to expand their socioeconomic network.

## Methods

### Data

This study uses data collected within the Matlab Health and Demographic Surveillance System which has been run by the International Centre for Diarrheal Disease Research, Bangladesh (ICDDR,B) since 1962 (Alam et al., 2017). Data used in this study were collected in 2010 from 944 women aged 20–65 years. The Health and Demographic Surveillance System includes a full population survey of over 200,000 people. For our subsample, equal numbers of women were drawn at random from three 15-year age categories (20–34, 35–49 and 50–64), allowing for better representation of older women in this growing population. Refusal rates were very low (under 5%) and were generally related to travel, personal illness or other immediate commitments (e.g. a daughter's wedding). During surveys, female, Bangla-speaking ICDDR,B data collectors – trained by Shenk and Alam – interviewed women at their homes. Women answered questions about each of their children including their children's marriages and questions regarding their natal families (e.g. family composition and parents’ education). Surveys also covered topics such as residence patterns, land ownership and inheritance, migration and fertility. For the purposes of this study, we included all female respondents who had ever married and any of their married daughters. The inclusion of women's married daughters had the benefit of expanding our sample size and increasing the number of relatively rare events for our outcome of interest (love marriages). This resulted in a working sample of 1598 women (886 focal women and 712 of their ever-married daughters).

This study received ethical approval from the Ethical Review Committee at ICDDR,B in Bangladesh (no. PR-09030) and the Campus Institutional Review Board at the University of Missouri in the USA (no. 1139478). According to these approvals, all participants gave written consent prior to taking part in the study.

### Variables

The primary variable of interest is marriage type, measured as arranged or love (including love and love–arranged). Other marriage variables captured women's age at marriage, and whether her spouse is a relative or not (0 = no, 1 = yes).

Women's status, or the individual characteristics likely to improve position on the marriage market, was measured in two ways: her education and information about her dowry. Education was measured in number of years of school completed. The dowry variable represents the total amount spent on dowry by women's parents and is reported in increments of 10,000 taka (~144 USD at the 2010 average exchange rate). Because dowries were given at various points in time, and the value of money fluctuates across time, this value is also adjusted for inflation to the value of 2010 Taka using the price of medium rice which may avoid overcorrection for inflation common with standard consumer price index (CPI) adjustments (although adjustment with standard CPI in this case yields similar results, see Lankes et al., 2022). To normalise the dowry variable, 1 was added to each value and then log transformed.

Husband's status was similarly estimated with a measure of years of education the husband had completed. Further, husbands’ occupations were classified by their social status (from low to high status occupations: 1 = agricultural and unskilled, 2 = semi-skilled, 3 = skilled labour and business, 4 = education-based and professional).

Information about women's fathers (occupational status and years of education) was used to both estimate the status of a woman's family of origin and their level of market integration. The family's level of market integration was measured via the father's occupation in two ways. Firstly, father's occupation was classified based on their level of market integration (as above). Secondly, father's occupation was classified as agricultural or not (0 = no, 1 = yes).

### Analysis

Data were analysed in Stata 15 and R version 4.0.3. We firstly used the complete sample of ever married women and their female children (*n* = 1598) to describe changes in marriages and other characteristics by age group, i.e. generational changes. We considered changes in types of marriages (love vs. arranged), marriages to relatives, education (own and husband's), and age at marriage.

Each prediction was then tested using regression-based models with clustered robust standard errors to account for non-independence of observations among mothers and daughters from the same family. Models were restricted to women and their daughters who were aged 50 and younger at the time of interview and had ever been married (*n* = 1340); this was done because in 2010 love marriages were extremely rare among women over 50 years old. Cases with missing data were excluded from analyses, resulting in variation in the final sample size across analyses. Each model had a different set of controls identified using directed acyclic graphs (DAGs; see below for details).

To test the first prediction that [P1] *women who had arranged marriages will have higher status spouses*, we ran two models with different outcome measures of spouse status: husband's education (linear regression) and occupational status (multinomial logistic regression. To test the second prediction that [P2] *being in an arranged marriage predicts younger ages at marriage*, we ran a linear regression model with marriage type predicting age at marriage. In addition to the first model, we ran a moderation analysis to explore the role of cousin marriage. Here, to predict age at marriage we included an interaction term between marriage type and cousin marriage. To test the third prediction that [P3] *being from a more market integrated family would result in a lower probability of being in an arranged marriage*, we ran two logistic regression models. In the first, the main predictor was the level of market integration of the woman's father's occupation, ranging from low to high. In the second set of models the primary predictor was a binary indicator of whether or not the woman's father's occupation was agricultural. Finally, to test the prediction that [P4] *education modifies the association between marriage type and odds of marrying a relative*, a logistic regression was run with marriage to a relative (yes/no) as the outcome and an interaction term between marriage type and educational attainment.

#### Directed acyclic graphs

**Figure 1.**
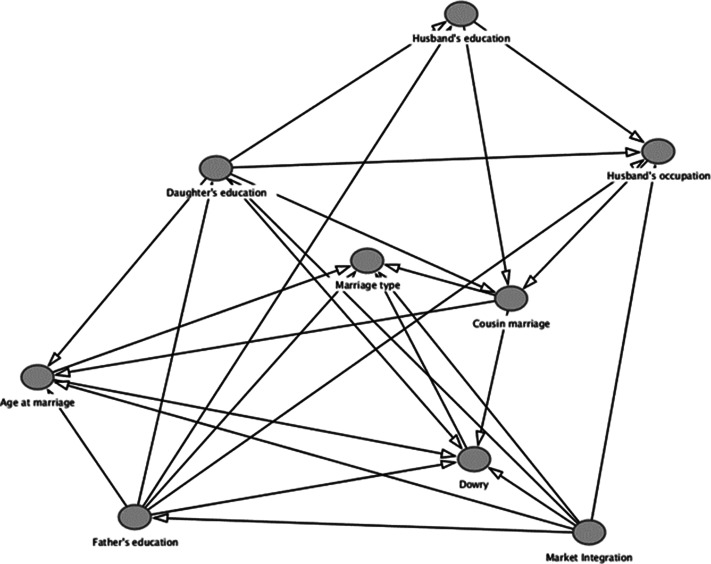
The directed acyclic graph (DAG) of all the predicted causal relationships between the variables included in the analysis. As this DAG underpins different model sets, variables have not been labelled as ‘exposures’ and/or ‘outcomes’, nor have the causal or biasing paths been highlighted.

We used DAGs to illustrate the predicted causal relationships between variables and identify confounders to adjust for using the dagitty package in R (Textor et al., 2016). Directed acyclic graphs are a form of graphical causal model which help to identify the cause–effect relationships we wish to model (Ankan et al., 2021). Based on our existing knowledge of causal relationships between variables, DAGs allow for the construction of a statistical model that can provide valid causal inference between the predicted relationships visualised in the DAG (McElreath, 2020). By including directional ties between variables, the use of DAGs allows the modelling of confounded (e.g. when A is associated with C because they are both causally influenced by B) and collider (e.g. when A and C both independently cause B) pathways. This permits the production of a statistical model that controls confounding relationships (e.g. closes the back door by including additional terms), and also avoids conditioning on the collider, which ensures the back doors remain closed (by not including these terms). The final DAG that underpins all models in this analysis is visualised in Figure 1, and is based on known relationships (as reviewed above) between key variables included in data collection, including age at marriage, cousin marriage, educational attainment of fathers, daughters and husbands, husbands’ occupational status, level of market integration, dowry amount and marriage type. As a result, each model (with a unique outcome and exposure) has different controls suggested by the minimally sufficient adjustment set for that specific causal relationship produced by the DAG, following confirmation of the implied conditional independencies (Ankan et al., 2021). The full process of creating and checking these DAGS can be found in the Supporting Information code, and full model outputs can be found in the Supporting Information results.

## Results

### Changes over time in marriage and education

Marriages that were not fully arranged are uncommon in this sample of women. Of all marriages for which information is available (*n =* 1598), 5.8% were not fully arranged, including 3.2% love–arranged marriages and 2.6% purely love marriages. Women who are in arranged marriages are older than women in love marriages (37 years vs. 31 years; *t* (d.f.) = 4.23 (1596); *p* < 0.001; Table 1). Half of all recorded love marriages were to women under 30 years old at the time of interview and three-quarters were to women aged 36 years and younger. Women in arranged marriages were married at younger ages than women in love marriages (17 vs. 18 years; *t* (d.f.) = −2.70 (1593); *p* = 0.007) and were less likely to be married to a relative compared with women in love marriages (19.8% of love marriages were to relatives vs. 9.0% of arranged marriages; *χ*^2^ = 10.44; *p* = 0.001). Women in arranged marriages were more likely to have had a dowry than women in love marriages (81.0% vs. 66.2%; *χ*^2^ = 8.96; *p* = 0.003) and this was largely driven by the fact that only half of women in purely love marriages had a dowry vs. 79.0% of those in love–arranged marriages (*χ*^2^ = 17.83; *p* < 0.001). Women's own education and that of their husbands was higher among women in love marriages compared with those in arranged marriages (women's mean years of education: 5.9 years vs. 5.0; *t* (d.f.) = −2.08 (1596); *p* = 0.038; husbands’ mean years of education: 6.9 vs. 5.7; *t* (d.f.) = −2.28 (1240); *p* = 0.022; Table 2). Women in love marriages were more likely to have a father (*χ*^2^ = 9.66; *p* = 0.022) and husband (*χ*^2^ = 9.72; *p* = 0.021) with an occupation classified as low–middle market integration (as compared with low, middle or high market integration) than women in arranged marriages. Women in love marriages were more likely to be married to a man with a non-agricultural occupation than women in arranged marriages (*χ*^2^ = 4.51; *p* = 0.034).

**Table 1.** Marriage characteristics by marriage type

	Total	Arranged	Love^a^	p^b^
N	1598	1506	92	
Age – mean (SD; min, max)	36.43 (11.95; 14, 67)	36.74 (12.03; 14, 67)	31.34 (9.12; 15, 57)	<0.001
Age at marriage – mean (SD; min, max)	17.40 (3.68; 7, 40)	17.34 (3.68; 7, 40)	18.41 (3.53; 12, 29)	0.007
Marriage type – n (%)				<0.001
Arranged	1506 (94.24)			
Love–arranged	51 (3.19)			
Love	41 (2.57)			
Spouse is relative – n (%)				0.001
No	1422 (90.06)	1349 (90.66)	73 (80.22)	
Yes	157 (9.94)	139 (9.34)	18 (19.78)	

a Includes love and love–arranged marriages.

b Chi-squared test if categorical; *t*-test if continuous.

**Table 2.** Woman's, husband's and family's status by marriage type

	Total	Arranged	Love^a^	p^b^
N	1598	1506	92	
Woman's status				
Education – mean (SD; min, max)	5.09 (3.93; 0, 16)	5.04 (3.92; 0, 16)	5.91 (3.98; 0, 15)	0.038
Had dowry, n (%)	1135 (80.27)	1090 (80.98)	45 (66.18)	0.003
Dowry – gross total in 10,000 takas (raw)^c^ – mean (SD; min, max)	23.10 (29.94; 0, 265)	23.20 (29.97; 0, 265)	20.89 (29.30; 0, 158)	0.613
Dowry – gross total in 10,000 takas (log)^c^ – mean (SD; min, max)	2.58 (1.12; 0, 6)	2.59 (1.13; 0, 6)	2.48 (1.11; 0, 5)	0.542
Husband's status				
Husband's education – mean (SD; min, max)	5.74 (4.49; 0, 16)	5.67 (4.47; 0, 16)	6.94 (4.72; 0, 16)	0.022
Occupational status – husband, n (%)				0.283
Agricultural and unskilled	387 (24.40)	357 (23.90)	30 (32.61)	
Semi-skilled labour	424 (26.73)	403 (26.97)	21 (22.83)	
Skilled labour and business	290 (18.28)	276 (18.47)	14 (15.22)	
Education-based and professional	485 (30.58)	458 (30.66)	27 (29.35)	
Family of origin – status and market integration				
Father's education – mean (SD; min, max)	3.53 (4.05; 0, 16)	3.57 (4.07; 0, 16)	2.96 (3.73; 0, 16)	0.161
Market dependency – father's occupation, n (%)				0.022
Low	733 (46.07)	694 (46.30)	39 (42.39)	
Low–middle	186 (11.69)	166 (11.07)	20 (21.74)	
Middle–high	264 (16.59)	251 (16.74)	13 (14.13)	
High	408 (25.64)	388 (25.88)	20 (21.74)	
Agricultural occupation – father, n (%)				0.220
Non-agricultural	874 (54.69)	818 (54.32)	56 (60.87)	
Agricultural	724 (45.31)	688 (45.68)	36 (39.13)	

a Includes love and love–arranged marriages.

b Chi-squared test if categorical; t-test if continuous.

c Among those who received any dowry.

**Figure 2.**
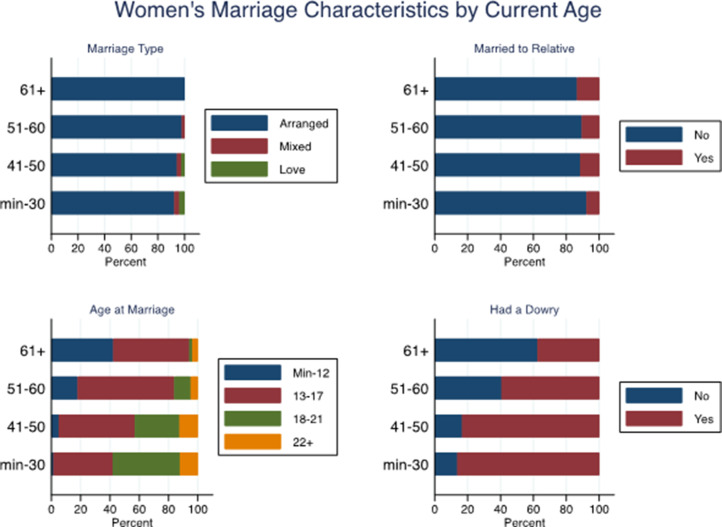
Women's marriage type, age at marriage, marriage to relative, and education by current age (*n* = 1598).

Just as age is a key correlate of marriage type, age also correlates with other characteristics of women, their marriages, their husbands and fathers. In particular we find that age at marriage was higher among younger women (Figure 2). Some 42% of women aged over 61 married at ages 12 years or below and 1.9% at ages 18–21 years compared with women aged 30 years and younger, among whom 1.4% married at age 12 and younger and 45.8% at ages 18–21 years (*χ*^2^ = 282.3; *p* < 0.001). Having had a dowry was also more common among younger women; while 38% of women aged 61 and above had a dowry, 86% of women aged 30 and under had a dowry (*χ*^2^ = 110.5; *p* < 0.001).

**Figure 3.**
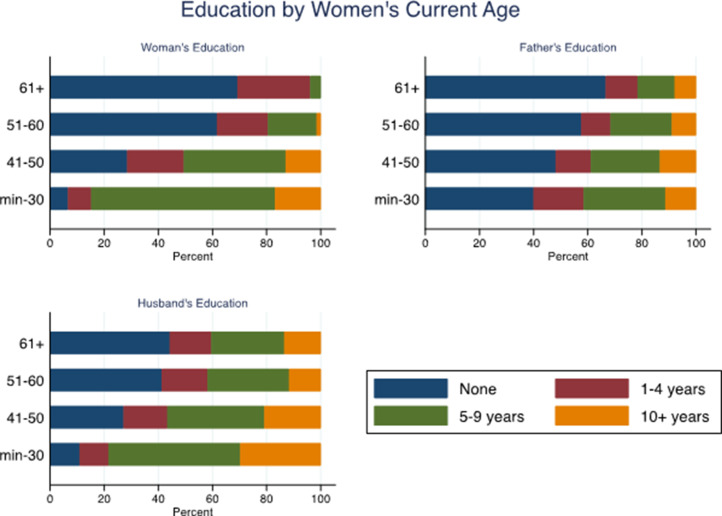
Women's father's and husband's years of education by women's current age.

Younger women had more years of education compared with older women, as did their husbands and fathers. While 69% of women aged 61 and above had no education, only 6.7% of women aged 30 years and under had no education (*χ*^2^ = 439.9; *p* < 0.001). Women who were older also were also more likely than younger women to have a father with no education (*χ*^2^ = 36.8; *p* < 0.001) or to be married to a man with no education (*χ*^2^ = 112.0; *p* < 0.001) (Figure 3).

### Prediction testing

From here we limit our sample to women aged under 50 years old as there was a lack of variation in marriage type in the sample older than 50 years.

#### Prediction 1: arranged marriage is positively associated with husband status

We find no evidence that women in arranged marriages were more likely to obtain husbands with either more education (*β* = −0.718, 95% confidence interval, CI [−1.546, 0.109], *n =* 961; Supporting Information, Table 1.1.1) or occupational status (low status relative risk ratio, RRR = 1.066, 95% CI [0.524,2.169]; unskilled labour RRR = 0.1.279, 95% CI [0.616, 2.656]; skilled labour RRR = 1.034, 95% CI [0.435, 2.456], relative to education-based employment, *n* = 1308; Supporting Information, Table 1.2.1) when controlling for marriage to a relative, daughter's education, father's education and level of market integration as determined by father's occupation. We found more evidence to support the role of women's status, as both women's (*β* = 0.703, 95% CI [0.643, 0.763], *n =* 977; Supporting Information, Table 1.1.2) and fathers’ educational attainment (*β* = 0.458, 95% CI [0.385, 0.531], *n =* 973; Table 1.1.3) was positively associated with the women's husbands’ educational attainment. Likewise, for husbands’ occupation, we do not find evidence that arranged marriages are predictive of a husband's occupational status (Supporting Information, Table 1.2.1), but we do find that a daughter's education predicts her husband having an education-based occupation (Supporting Information, Table 1.2.2).

#### Prediction 2: arranged marriage predicts a younger age at marriage

We do not find evidence for a relationship between marriage type and age at marriage, controlling for cousin marriage, daughter's and father's education and market integration (Supporting Information, Table 2.1). The beta estimate, however, is in the expected direction (*β* = −0.674, 95% CI [−1.374, 0.081], *n* = 1303), suggesting a weak negative relationship between arranged marriage and age at marriage. We ran an additional model with an interaction between arranged marriage and cousin marriage to predict age at marriage. While the interaction term was non-significant (*β* = −0.789, *p =* 0.417, 95% CI −2.697, 1.119]; Supporting Information, Table 2.2), this analysis showed that compared with an arranged, non-cousin marriage, an arranged cousin marriage occurred 0.871 years earlier (*p =* 0.005, 95% CI [−1.477, −0.266, *n =* 1311). As a result, the average age of marriage for a cousin-arranged marriage was 17.05 years, compared with 18.34 for a cousin non-arranged marriage, or 18.43 for a non-arranged, non-cousin marriage and 17.92 for a non-cousin, non-arranged marriage.

#### Prediction 3: higher levels of market integration are associated with a lower likelihood of having an arranged marriage

We do not find support for the prediction that level of market integration at the family level, as measured by father's occupation, is an important predictor of marriage type (Supporting Information, Table 3.1 and Supporting Information, Table 3.2). While compared with low levels of market integration, low–middle market integration (non-food-production occupations) was significantly associated with a decrease in odds of arranged marriage (odds ratio, OR = 0.499, 95% CI [0.282, 0.882], *n* = 1333), this finding did not hold among either middle–high or high levels of market integration, which demonstrated (non-significant) increased odds of arranged marriages. Furthermore, a father's occupation being agriculture/food production, as compared with not being involved in agriculture or food production, was not associated with marriage type (OR = 0.793, 95% CI [0.552, 1.381], *n* = 1340).

#### Prediction 4: women with lower educational attainment who enter love marriages will be more likely to marry a relative

**Figure 4.**
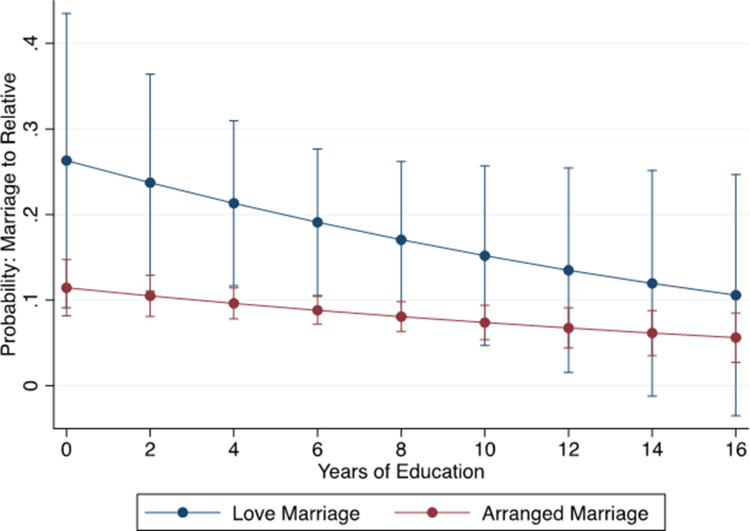
Predicted probability of marrying a relative and 95% confidence intervals by marriage type and years of education based on model 4.

In line with our predictions, being in an arranged marriage (controlling for daughter's and father's education and market integration) was associated with a decrease in the odds of cousin marriage (OR = 0.392, 95% CI [0.212, 0.726], *n =* 1314; Supporting Information, Table 4.1). We predicted that this effect would be dependent on exposure to non-relatives (via education), and therefore ran an additional interaction between marriage type and education attainment. While the confidence interval for an interaction between marriage type and education overlaps with 1 (OR = 0.364, 95% CI [0.885, 1.170], *n =* 1314; Supporting Information, Table 4.2), the direction of the effect is as predicted (Figure 4). That is, the trend in the data indicates that women with lower education who were in love marriages were more likely to marry a cousin than women with more education or women who were in arranged marriages regardless of education level.

## Discussion

Our findings confirm high rates of arranged marriage in Matlab, Bangladesh. Love marriages were concentrated among younger women, including both love–arranged marriages, in which parents facilitate the marriage of daughters to a spouse chosen by the daughter, and purely love marriages, which take place without parental facilitation. Love–arranged marriages are more common than purely love marriages for older women while among younger women the split between love–arranged and purely love marriages was more even. It is probable, in this context, that many marriages that are not fully arranged are still taking place with some parental involvement evidenced by the fact that even among marriages classified as purely love marriages (that is, excluding love–arranged marriage), half took place with a dowry. This suggests at least some level of parental involvement in the marriage, either by parents coming to agree with their daughter's choice over time or by them choosing to save face by formally approving the marriage and giving a dowry to socially legitimise the marriage. We find that marriage type is a poor predictor of spouse characteristics, possibly because in practice there are limited socioeconomic distinctions between arranged and love marriages – and particularly between arranged and love–arranged marriages. Rather, measures of the family's status or bargaining power (father's education) and women's own educational status are more important predictors of spouse status. This supports the idea that regardless of whether a marriage is arranged, parents can improve their daughters’ chances on the marriage market by investing directly in their daughter through her education.

There is some evidence that arranged marriages occur at younger ages than non-arranged marriages (Prediction 2), in line with hypotheses derived from parent-offspring conflict theories (Schaffnit, Hassan et al., 2019; Corno and Voena, 2016). However, this result is largely accounted for by the small number of arranged marriages to relatives, which occurred significantly earlier than arranged marriage to non-relatives. While this is contrary to our expectations, this result is easy to understand. Given existing family ties, marriages to relatives are likely to be easier to arrange because the proposed spouses have pre-existing relationships to ease the process; they may even be arranged far in advance of the actual wedding. Our results did highlight that marriages to relatives were much more likely to be love marriages, rather than arranged marriages (Prediction 4). This is in line with previous research in Matlab and South Asia, which found that given restricted interactions with non-relatives of the opposite sex, relatives have far more occasion to interact and fall in love (Ghimire et al., 2006; Shenk, Towner et al., 2016). However, we saw no evidence, that love marriages occurred at younger ages among relatives. It may be the case then that, compared with arranged marriages with non-relatives, relatedness did not necessarily speed up the process of prospective spouses falling in love and deciding to marry.

**Figure 5.**
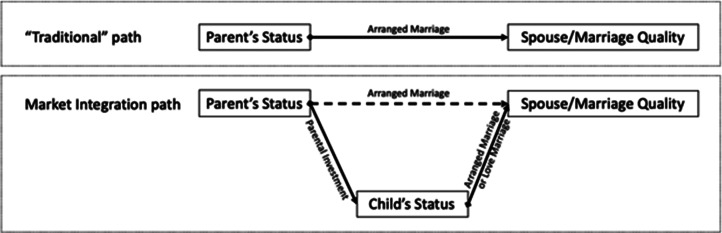
Visualisation of traditional and market integrated pathways to a high-quality spouse/marriage.

We did not find any evidence that family-level indicators of market integration (estimated by women's fathers’ occupation) predict the type of marriage. However, the rise of non-arranged marriages does appear to be linked to several aspects of market integration on a societal level, although this finding is descriptive. Market integration is clearly linked to increasing parental investments in South Asia, particularly in education, but also to investment in dowry (Shenk, 2004; Srinivas, 1984); we see this in our own data with younger women being more likely to have had a dowry, having higher education and being married to men with higher education. We argue that such investments, especially in daughter's education, have created a situation in which love marriages are now possible where they were not in the past. Increasing parental investment owing to market integration has led to increasing female education. This results in delayed marriage for those women participating in education, which can be seen in increasing ages at marriage. Together, higher levels of education and older ages at marriage mean that women now have (a) greater bargaining power than in the past, (b) more opportunities to meet a husband outside of their parents’/family's network and (c) greater ability to attract a high-status spouse owing to their own status rather than having to rely on their father's or family's status. Together this creates an environment where non-arranged marriages (including many approved by parents) may become more common despite a deeply entrenched arranged marriage system. This suggests that we are currently seeing a shift within Matlab from the ‘traditional’ path to ensuring high spouse/marriage quality to a ‘market integration’ path (Figure 5). In the former, parents use their status to arrange advantageous marriages, in part through dowry. In the latter, parents invest heavily in their daughters (e.g. through education), which means that daughters are in a position to attract a high-status spouse, either on their own (through their education) or with parental assistance (through their dowry).

There is some evidence that the two key parental investments that allow for the new ‘market integrated’ path to marriage in this setting – investment in dowry and education – may trade off against each other (Goel & Barua, 2021; Walker et al., 2008; Shenk, 2004; Shahidul, 2014), but generally speaking parents invest significantly in both. Yet our results show that while investment in a daughter's education is a significant predictor of her husband's status, investment in dowry had only a small, positive and non-significant relationship with husband's status. This is probably because education has many collateral effects relevant to arranged marriage. For example, more years of education are accompanied by delays to marriage, increased human capital for women, an increased ability to invest in her own children's human capital (Shenk, 2004; Shenk, Towner et al., 2016; Goel & Barua, 2021), and additional opportunities to meet men who match her socioeconomic status. In contrast, dowry represents a major financial investment that is associated with spouse education – a measure of quality – but may have relatively few collateral effects (although see Shenk, 2007 for a possible positive effect on child outcomes). This may also be because dowry is paid by parents and thus is linked to parental resources and status and/or spouse resources and status, rather than representing an independent characteristic of the bride.

In fact, it is not clear whether dowry should go on the upper or lower path of Figure 5. On the one hand, parents typically pay dowry and thus it is a strong reflection of parental status and resources. On the other hand, dowries accompany daughters into a marriage. Dowry thus serves as both a direct and indirect investment in the status and resources of the daughter and her children, increases women's bargaining power in her marital family (Shenk, 2007; Makino, 2019; Goel & Barua, 2021) and can be classified as a form of parental investment. In all likelihood, dowries represent aspects of parents’, women's and husbands’ status, thus such interpretations may be hard to distinguish.

While family-level market integration does not seem to affect marriage patterns, at least using our simple measures, we do find that an individual-level marker of market integration, education, clearly affects individual marriage outcomes. We also suggest that society-level changes in education, and the accompanying strengthened bargaining power of women with higher education (Ikhar et al., 2022), leads to altered marriage opportunities for everyone, even those who themselves do not have high education. This can be seen in the interaction between education and marriage type; women with low education levels are able to participate in love marriages despite their young age and lack of (relative) economic power *because a new pathway to marriage has been opened within their society*. Owing to constraints around who young unmarried girls and women interact with, these marriages are typically to male relatives with whom they are able to interact more freely. Yet we see that the opportunity for non-arranged marriages, which arises partially owing to increasing women's education, is available across all levels of education. This pattern also echoes patterns of prestige-biased transmission (Boyd & Richerson, 1985) in which adoption of behaviours by higher-status members of a society may transmit new social norms or allow for the adoption of new behaviours by others.

### Limitations and future research

This study benefits from a rich dataset from a single population with a large sample size. One of the strengths of this study was having access to data on marriage from two generations of women. This allowed us to both increase our sample size and also add instances of a relatively rare outcome (non-arranged marriages). Even so, the outcome remains very rare in this population which may limit the generalisability and interpretability of the statistics we present. Our largely null results may be the product of a Type II error, and future studies should endeavour to work with larger samples of love marriages to replicate the results presented here. A trade-off in the benefits of including data from focal women's daughters was that we lost various measures of childhood socioeconomic position and parents’ status that were only available for focal women but not their daughters. Future work would benefit from using data which allows for broader measures of family status and spouse quality. Such measures could then be used to build path models that in turn may help to explain the primary drivers and consequences of arranged marriage, as well as the potential moderating effects of level market integration at the society and individual level.

It is also important to note that the father's occupation data used to proxy market integration is measured at the time of the survey (or at the time that he retired), but the marriages of women in our sample happened before this time. While based on ethnographic knowledge, we have reason to believe that occupations are relatively stable across the lifespan in this part of Bangladesh, it is nonetheless likely that the effect of market integration as measured here may be underestimated. While we do have qualitative data on related topics (e. g. Shenk, Towner et al., 2016), our understanding of arranged marriage in Matlab would benefit from further use of qualitative methods (e.g. Baraka et al., 2022; Akurugu et al., 2022) to clarify the connections between market integration and marriage decisions.

It is also important to note that, as the instances of love marriages are higher among younger women, our findings speak more to the younger generation than to the older. Moreover, in the time since these data were collected, the marriage system in Matlab has continued to evolve. Since 2010 the digital era has come to Matlab, and mobile phones – particularly smart phones with connections to messaging apps and social media – have become common and heavily used among young people, especially those who are wealthier, more educated and studying in high school or college. Through their phones they have more contact with other young people, including both locals but also those who are unknown or distant who they would not have come into contact with in the past. ICDDR,B fieldworkers in Matlab have observed an increase in the incidence of love marriage in general, including many love marriages among younger people, contributing to a surprising trend towards lower ages at marriage in the area in the last decade – and ICDDR,B internal data show that in 2018 arranged–love and love marriage together accounted for around 15% of recent marriages with an increasing trend.

In this paper we focused on the arranged marriage of daughters, in part because arranged marriages are more common for daughters than for sons. Future research in Matlab would do well to consider within-family tradeoffs in distributing resources between sons and daughters. Parents in this context, as in much of South Asia, attempt to weigh investments in daughter's status (e.g. education) against investments in her dowry, wanting to strike the right balance to attract an advantageous marriage (Goel & Barua, 2021; Shahidul, 2014). The same is not true for sons since he does not take a dowry into marriage with him and thus investments can be channeled fully into increasing his status. This may create conflicts within families as they weigh the various tradeoffs in within-child and between-child investments to optimise benefits for the family unit.

## Conclusion

Marriages arranged by parents with accompanying dowry payments remain the norm in Matlab, Bangladesh. The area, however, is in the midst of rapid market integration – a process which has been associated with changes in, and the weakening of, arranged marriage systems in other settings (Reed, 2019; Allendorf & Pandian, 2016). While we do not find support for the idea that family-level markers of market integration are associated with marriage type in this sample, we argue that on a societal level market integration has led to changes which have altered key dynamics in the marriage market in the region. These changes, seen in parental investment strategies and resultant improvements in children's bargaining power, have opened a new path to entering a marriage in Matlab, one which relies on women using their own status, gained through intensive parental investments in their education, to help in the negotiation of advantageous marriages. We argue that increasing female education, a key accompaniment of market integration and major form of parental investment in market integrating societies, is particularly important in opening this novel pathway owing to its far-reaching collateral effects relevant to marriage processes. Over time we expect that these forces will result in marriage decisions increasingly being made jointly between parents and children, as has been shown in urban India (Reed, 2019; Allendorf & Pandian, 2016) and other related contexts.

## Data Availability

The data that support the findings of this study are not currently publicly available owing to IRB limitations. Data are available from MS and subject to a data use agreement.

## Research transparency and reproducibility

The code used to run the statistical models in this paper are available as supplementary files.

## Supplementary material

For supplementary material accompanying this paper visit https://doi.org/10.1017/ehs.2022.54. click here to view supplementary material
