# Supplementary material for: The impact of market integration on arranged marriages in Matlab, Bangladesh
Source: Evol Hum Sci. 2022 Dec 12;5:e5. doi: 10.1017/ehs.2022.54 (PMC10426007; doi:10.1017/ehs.2022.54)
Supplement: Supplementary file 1 [file S2513843X22000548sup.zip › S2513843X22000548sup002.pdf]

Supplementary Materials

Below are the full models used to test each of the four predictions made in the main paper. In each model, controls were chosen based on the minimally sufficient adjustment set identified by the DAG (see methods).

1. Prediction 1: Arranged marriage is positively associated with husband status

1.1. Outcome: Husband's Education

Table 1.1.1 Main model for prediction 1, testing for the relationship between arranged marriage and husband's level of education.
Model outcome: Husband's Education
Independent variables: Arranged marriage + married to relative + daughter's education + father's education + father's occupational status

Coefficients:	Beta	Robust Standard Error	t	p	95% Confidence Interval	
(Intercept)	2.801	0.500	5.596	0.000	1.819	3.783
Marriage arranged (ref: love marriage)	-0.718	0.422	-1.703	0.089	-1.546	0.109
Married to spouse (ref: no)	-0.257	0.363	-0.707	0.480	-0.969	0.455
Daughter's education	0.692	0.030	22.744	0.000	0.632	0.752
Father's Education	0.176	0.032	5.445	0.000	0.113	0.240
Father's occupation: Education-based (ref: business)	-0.205	0.330	-0.620	0.535	-0.853	0.443
Father's occupation: Non-food production (ref: business)	-0.323	0.503	-0.643	0.521	-1.311	0.664
Father occupation: Primary food production (ref: business)	-0.358	0.296	-1.210	0.227	-0.940	0.223

Residual standard error: 3.182 on 953 degrees of freedom (379 observations deleted due to missingness)
Multiple R-squared: 0.4919, Adjusted R-squared: 0.4882
F-statistic: 131.8 on 7 and 953 DF, p-value: < 2.2e-16

Table 1.1.2 Model with daughter's education predicting husband's education.
Model outcome: Husband's Education
Independent variables: daughter's education + father's education

Coefficients:

	Beta	Robust Standard Error	t	p	95% Confidence Interval	
(Intercept)	1.792	0.203	8.840	0.000	1.394	2.190
Daughter's education	0.703	0.030	23.137	0.000	0.643	0.763
Father's education	0.173	0.030	5.852	0.000	0.115	0.231

Residual standard error: 3.174 on 974 degrees of freedom (363 observations deleted due to missingness)
Multiple R-squared: 0.4905, Adjusted R-squared: 0.4894
F-statistic: 468.8 on 2 and 974 DF, p-value: < 2.2e-16

Table 1.1.3 Model with father's education predicting husband's education.
Model outcome: Husband's Education
Independent variables: father's education + father's occupational status

Coefficients:

	Beta	Robust Standard Error	t	p	95% Confidence Interval	
(Intercept)	5.182	0.361	14.353	0.000	4.473	5.890
Father's education	0.458	0.037	12.238	0.000	0.385	0.531
Father's occupation: Education-based (ref: business)	-0.109	0.434	-0.251	0.802	-0.960	0.742
Father's occupation: Non-food production (ref: business)	-1.177	0.560	-2.101	0.036	-2.277	-0.077
Father occupation: Primary food production (ref: business)	-0.784	0.392	-1.998	0.046	-1.554	-0.014

Residual standard error: 3.963 on 968 degrees of freedom (367 observations deleted due to missingness)
Multiple R-squared: 0.2066, Adjusted R-squared: 0.2033
F-statistic: 63.02 on 4 and 968 DF, p-value: < 2.2e-16

Table 1.1.4 Model with log dowry predicting husband’s education.
Model outcome: Husband's Education
Independent variables: log dowry + {father's education + daughter's education + father's occupational status}

Coefficients:

	**Beta**	**Robust Standard Error**	**t**	**p**	**95% Confidence Interval**	
(Intercept)	1.916	0.389	4.932	0.000	1.154	2.679
Dowry (log)	0.144	0.096	1.503	0.133	-0.044	0.333
Father’s education	0.166	0.034	4.847	0.000	0.099	0.233
Daughter’s education	0.671	0.033	20.053	0.000	0.606	0.737
Father’s occupation: Education-based (ref: business)	-0.166	0.353	-0.470	0.638	-0.859	0.527
Father’s occupation: Non-food production (ref: business)	-0.270	0.557	-0.484	0.629	-1.364	0.824
Father occupation: Primary food production (ref: business)	-0.364	0.315	-1.158	0.247	-0.982	0.253
Married to relative (ref: no)	-0.210	0.400	-0.525	0.599	-0.996	0.575

Residual standard error: 3.205 on 850 degrees of freedom (482 observations deleted due to missingness)
Multiple R-squared: 0.4798, Adjusted R-squared: 0.4755
F-statistic: 112 on 7 and 850 DF, p-value: < 2.2e-16

1.2. Outcome: Husband’s Occupational Status

Table 1.2.1 Main model for prediction 1, testing for the relationship between arranged marriage and husband’s occupational status.
Model outcome: Husband's Occupational Status
Independent variables: Arranged marriage + married to relative + daughter's education + father's education + level of market integration
Multinomial logistic regression Number of obs = 1,308
Wald chi2(21) = 689.09
Prob > chi2 = 0.0000
Log pseudolikelihood = -1254.9719 Pseudo R2 = 0.2985
(Std. Err. adjusted for 822 clusters in resp_id_questionaire)

Husband’s Occupational Status	**Relative Risk Ratio**	**Robust Standard Error**	**z**	**P>z**	**95% Confidence Interval**	
**Low**						
Marriage arranged (ref: love marriage)	1.066	0.386	0.180	0.859	0.524	2.169
Married to spouse (ref: no)	1.190	0.386	0.540	0.591	0.630	2.249
Daughter’s education	0.832	0.025	-6.230	0.000	0.785	0.882
Father’s education	0.936	0.036	-1.740	0.081	0.869	1.008
Father’s occupation (ref: business)						
Non-food production	10.664	4.532	5.570	0.000	4.637	24.530
Business	0.482	0.158	-2.230	0.026	0.254	0.917
Education-based	0.091	0.026	-8.520	0.000	0.053	0.158
_cons	3.978	1.569	3.500	0.000	1.836	8.618
**Unskilled_labor**						
Marriage arranged (ref: love marriage)	1.279	0.477	0.660	0.508	0.616	2.657
Married to spouse (ref: no)	0.747	0.269	-0.810	0.419	0.368	1.515
Daughter’s education	0.933	0.027	-2.390	0.017	0.881	0.988
Father’s education	0.999	0.030	-0.030	0.978	0.942	1.059
Father’s occupation (ref: business)						
Non-food production	0.520	0.284	-1.200	0.231	0.178	1.516
Business	0.212	0.066	-5.010	0.000	0.116	0.389

Education-based	0.031	0.009	-12.630	0.000	0.018	0.054
_cons	3.490	1.450	3.010	0.003	1.547	7.878
**Skilled_labor**						
Marriage arranged (ref: love marriage)	1.034	0.457	0.070	0.940	0.435	2.457
Married to spouse (ref: no)	0.725	0.272	-0.860	0.391	0.348	1.511
Daughter’s education	1.001	0.031	0.030	0.976	0.942	1.064
Father’s education	0.947	0.027	-1.910	0.057	0.895	1.002
Father’s occupation (ref: business)						
Non-food production	2.431	1.269	1.700	0.089	0.874	6.762
Business	10.384	2.884	8.420	0.000	6.024	17.898
Education-based	0.378	0.099	-3.700	0.000	0.226	0.633
_cons	0.515	0.264	-1.290	0.196	0.189	1.407
**Education_based | (base outcome)**						

Table 1.2.2 Model with daughter's education predicting husband's occupational status.
Model outcome: Husband's Occupational Status
Independent variables: daughter's education + father's education

Multinomial logistic regression — Number of obs = 1,323; Wald chi2(6) = 140.99; Prob > chi2 = 0.0000; Pseudo R2 = 0.0627

Log pseudolikelihood = -1697.3342

(Std. Err. Adjusted for 833 clusters in resp_id_questionaire)

Husband's Occupational Status Education_based	**Relative Risk Ratio** |	**Robust Standard Error** (base	**z** outcome)	**P**	**95% Confidence Interval**	
**Low**						
Daughter's education	0.833	0.021	-7.360	0.000	0.793	0.874
Father's education	0.840	0.028	-5.300	0.000	0.786	0.895
_cons	3.975	0.727	7.540	0.000	2.777	5.690
**Unskilled_labor**						
Daughter's education	0.931	0.023	-2.950	0.003	0.887	0.976
Father's education	0.899	0.023	-4.200	0.000	0.856	0.945
_cons	2.033	0.391	3.690	0.000	1.395	2.964
**Skilled_labor**						
Daughter's education	0.990	0.027	-0.360	0.720	0.938	1.045
Father's education	0.902	0.023	-4.030	0.000	0.858	0.948
_cons	0.998	0.222	-0.010	0.992	0.645	1.543

Table 1.2.3 Model with father's education predicting husband's occupational status.
Model outcome: Husband's Occupational Status
Independent variables: father's education + level of market integration

Multinomial logistic	regression		Number of obs		= 1,320	=1,340
			Wald chi2(12)		627.92	
			Prob > chi2		0	
Log pseudolikelihood	-1306.3258		Pseudo R2		0.277	=0.2772

(Std. Err. Adjusted for 831 clusters in resp_id_questionaire)

Husband's Occupational Status	**Relative Risk Ratio**	**Robust Standard Error**	**z**	**P**	**95% Confidence Interval**	
**Education_based**	|	(base	outcome)			
**Low**						
Father's education	0.882	0.030	-3.720	0.000	0.825	0.942
Father's occupation (ref: business)						
Non-food production	10.724	4.376	5.810	0.000	4.820	23.862
Business	0.501	0.161	-2.160	0.031	0.267	0.939
Education-based	0.092	0.025	-8.950	0.000	0.055	0.155
_cons	1.992	0.345	3.980	0.000	1.419	2.796
**Unskilled_labor**						
Father's education	0.979	0.027	-0.760	0.448	0.928	1.033
Father's occupation (ref: business)						
Non-food production	0.537	0.292	-1.140	0.253	0.185	1.559
Business	0.223	0.069	-4.86	0	0.121	0.408
Education-based	0.031	0.009	-12.53	0	0.018	0.054
_cons	*2.952*	0.466	6.850	0.000	2.166	4.023
**Skilled_labor**						
Father's education	0.956	0.025	-1.670	0.095	0.908	1.008
Father's occupation (ref: business)						
Non-food production	2.492	1.292	1.76	0.078	0.902	6.886
Business	10.357	2.851	8.49	0	6.038	17.764
Education-based	0.404	0.103	-3.56	0	0.245	0.666
_cons	0.504	0.100	-3.44	0.001	0.341	0.744

2. Prediction 2: Arranged marriage predicts a younger age at marriage

Table 2.1 Model to test prediction 2, with no interaction between arranged marriage and marriage to relative.
Model outcome: Age at Marriage
Independent variables: Arranged marriage + married to relative + daughter's education + father's education + father's occupational status

Coefficients:

	**Beta**	**Robust Standard Error**	**t**	**p**	**95% Confidence Interval**	
(Intercept)	16.405	0.472	34.749	0.000	15.479	17.331
Marriage arranged (ref: love marriage)	*-0.647*	*0.371*	*-1.743*	*0.082*	*-1.374*	*0.081*
Married to spouse (ref: no)	-0.779	0.295	-2.644	0.008	-1.357	-0.201
Daughter's education	0.352	0.031	11.258	0.000	0.290	0.413
Father's education	-0.041	0.029	-1.427	0.154	-0.097	0.015
Father's occupation: Education-based (ref: business)	-0.076	0.347	-0.219	0.827	-0.756	0.604
Father's occupation: Non-food (ref: business)	0.713	0.382	1.867	0.062	-0.036	1.463
Father's occupation: Primary (ref: business)	0.462	0.321	1.437	0.151	-0.169	1.092

Residual standard error: 3.222 on 1303 degrees of freedom (29 observations deleted due to missingness)
Multiple R-squared: 0.1379, Adjusted R-squared: 0.1333
F-statistic: 29.78 on 7 and 1303 DF, p-value: < 2.2e-16

Table 2.2 Model to test prediction 2, with interaction between arranged marriage and marriage to relative.
Model outcome: Age at Marriage
Independent variables: Arranged*Marriage to Cousin + daughter's education + father's education + father's occupational status

Coefficients:

	**Beta**	**Robust Standard Error**	**t**	**p**	**95% Confidence Interval**	
(Intercept)	16.265	0.506	32.173	0.000	15.273	17.256
Marriage arranged (ref: love marriage)	-0.506	0.410	-1.233	0.218	-1.311	0.299
Married to spouse (ref: no)	-0.082	0.926	-0.089	0.929	-1.899	1.734
Daughter’s education	0.352	0.031	11.277	0.000	0.291	0.413
Father’s education	-0.041	0.028	-1.428	0.153	-0.097	0.015
Father’s occupation: Education-based (ref: business)	-0.068	0.347	-0.197	0.844	-0.750	0.613
Father’s occupation: Non-food (ref: business)	0.728	0.382	1.906	0.057	-0.021	1.477
Father’s occupation: Primary (ref: business)	0.468	0.322	1.453	0.146	-0.164	1.099
Arranged marriage (ref: love) * Marriage to relative (ref: no)	-0.789	0.972	-0.812	0.417	-2.697	1.119

Residual standard error: 3.223 on 1302 degrees of freedom (29 observations deleted due to missingness)
Multiple R-squared: 0.1384, Adjusted R-squared: 0.1331
F-statistic: 26.14 on 8 and 1302 DF, p-value: < 2.2e-16

3. Prediction 3: Higher levels of market integration is associated with lower likelihood of having an arranged marriage

Table 3.1 Model to test prediction 3, with level of family market integration predicting marriage type (arranged marriage).
Model outcome: Arranged Marriage
Independent variable: market integration

Coefficients:

	**Odds Ratio**	**Robust Standard Error**	**t**	**p**	**95% Confidence Interval**	
(Intercept)	14.342	0.181	14.749	0.000	10.067	20.432
Market integration: High (ref: low)	1.259	0.299	0.769	0.442	0.700	2.263
Market integration: Low-Middle (ref: low)	0.499	0.291	-2.392	0.017	0.282	0.882
Market integration: Middle-High (ref: low)	1.356	0.355	0.860	0.390	0.677	2.718

Null deviance: 648.40 on 1332 degrees of freedom
Residual deviance: 639.19 on 1329 degrees of freedom (7 observations deleted due to missingness)
AIC: 647.19

Table 3.2 Model to test prediction 3, with participation in agriculture predicting marriage type (arranged marriage).
Model outcome: Arranged Marriage
Independent variable: Agriculture

Coefficients:

	**Odds Ratio**	**Robust Standard Error**	**t**	**p**	**95% Confidence Interval**	
(Intercept)	15.400	0.189	14.469	<2e-16	10.633	22.304
Non-agricultural occupation (ref: agricultural)	0.874	0.234	-0.578	0.5631	0.552	1.381

Null deviance: 649.35 on 1339 degrees of freedom
Residual deviance: 648.99 on 1338 degrees of freedom
AIC: 652.99

4. Prediction 4: Women with lower educational attainment who enter love marriages will be more likely to marry a relative

Table 4.1 Model for prediction 4 with no interaction between arranged marriage and education.
Model outcome: Married to cousin
Independent variables: arranged + daughter's education + father's education + level of market integration

Coefficients:

	**Odds Ratio**	**Robust Standard Error**	**t**	**p**	**95% Confidence Interval**	
(Intercept)	0.330	0.343	-3.228	0.001	0.169	0.647
Arranged marriage (ref: love marriage)	0.392	0.315	-2.977	0.003	0.212	0.726
Daughter's education	0.925	0.029	-2.638	0.008	0.873	0.980
Father's education	1.013	0.028	0.466	0.641	0.959	1.070
Market Integration: High (ref: low)	1.574	0.236	1.921	0.055	0.991	2.500
Market Integration: Low-Middle (ref: low)	0.812	0.319	-0.654	0.513	0.434	1.517
Market Integration: Middle-High (ref: low)	0.770	0.306	-0.856	0.392	0.423	1.401

Null deviance: 821.33 on 1313 degrees of freedom
Residual deviance: 800.93 on 1307 degrees of freedom
(26 observations deleted due to missingness)
AIC: 814.93

Table 4.2 Model for prediction 4 with interaction between arranged marriage and education.
Model outcome: Married to cousin
Independent variables: arranged * daughter's education + father's education + level of market integration

	**Odds Ratio**	**Robust Standard Error**	**t**	**p**	**95% Confidence Interval**	
(Intercept)	0.358	0.487	-2.109	0.035	0.138	0.930
Love marriage (ref: arranged marriage)	0.357	0.497	-2.072	0.038	0.135	0.946
Daughter's education	0.912	0.070	-1.315	0.189	0.794	1.046
Father's education	1.013	0.028	0.465	0.642	0.959	1.070
Market integration: High (ref: low)	1.572	0.236	1.919	0.055	0.990	2.496
Market integration: Low-Middle (ref: low)	0.808	0.322	-0.662	0.508	0.430	1.519
Market integration: Middle-High (ref: low)	0.769	0.306	-0.857	0.391	0.423	1.401
Love marriage * Daughter's education	0.364	0.071	0.241	0.810	0.885	1.170

Null deviance: 821.33 on 1313 degrees of freedom
Residual deviance: 800.87 on 1306 degrees of freedom (26 observations deleted due to missingness)
AIC: 816.87
